# Abnormal Newborn Screening Resembling Carnitine Palmitoyltransferase 1a Deficiency in Three Patients With *COASY* Protein Associated Neurodegeneration

**DOI:** 10.1002/jmd2.70066

**Published:** 2026-02-26

**Authors:** Matthew Lynch, Sophie Manoy, Claire Murray, Geoff Wallace, Nolette Pereira, Ricky Price, Anita Inwood, Jim McGill, David Coman

**Affiliations:** ^1^ Queensland Lifespan Metabolic Medicine Service Queensland Children's Hospital Brisbane Australia; ^2^ Department of Neurosciences Queensland Children's Hospital Brisbane Australia; ^3^ School of Medicine The University of Queensland Brisbane Australia; ^4^ Department of Radiology Queensland Children's Hospital Brisbane Australia; ^5^ Newborn Screening Queensland The Royal Brisbane and Women's Hospital Brisbane Australia; ^6^ School of Nursing and Social Work The University of Queensland Brisbane Australia; ^7^ Department of Chemical Pathology The Royal Brisbane and Women's Hospital Brisbane Australia; ^8^ School of Medicine Griffith University Gold Coast Australia

**Keywords:** carnitine palmitoyltransferase 1a deficiency, *COASY* protein associated neurodegeneration, inborn error of metabolism, newborn bloodspot screening

## Abstract

*COASY* protein associated neurodegeneration is a rare, progressive autosomal recessive neuroferritinopathy due to pathogenic mutations in the *COASY* gene, coding for the mitochondrial located coenzyme A synthase. Clinical manifestations include seizures, progressive spasticity, dystonia, neuropathy, cognitive decline and neuropsychiatric abnormalities. Both foetal and childhood onset phenotypes are described. We report three patients with *COASY* protein associated neurodegeneration who were identified on newborn screening with a dried bloodspot acylcarnitine pattern consistent with carnitine palmitoyltransferase 1a deficiency, that is, an elevated ratio of free carnitine (C0) to the sum of palmitoylcarnitine (C16) and octanoylcarnitine (C18):[C0/(C16+C18)]. Two siblings, who died in infancy, displayed neurological features from birth, with magnetic resonance imaging of the brain displaying immature cortical sulcation, parenchymal atrophy and pontocerebellar hypoplasia. The third patient presented with global developmental delay, pyramidal signs and seizures with brain magnetic resonance imaging at age 15 months demonstrating a thin corpus callosum, symmetric diffusion restriction throughout the basal ganglia and evidence of deposition in the globus pallidus. This report demonstrates that phenotypes of *COASY* protein associated neurodegeneration should be included in the differential diagnosis of dried blood spot acylcarnitine pattern suggestive of carnitine palmitoyltransferase 1a deficiency and may represent new potential for early diagnosis.

## Key Points

- We report three patients with COASY protein associated neurodegeneration (CoPAN) who were identified on newborn screening with a dried bloodspot (DBS) acylcarnitine pattern consistent with carnitine palmitoyltransferase 1a (CPT1) deficiency.
- CoPAN should be included in the differential diagnosis of dried blood spot acylcarnitine pattern suggestive of CPT1 deficiency.
- A DBS pattern of CPT1 deficiency with a normal plasma acylcarnitine profile is suggestive of CoPAN and can be detected prior to the onset of neurological manifestations, identifying new potential for early diagnosis.

## 1 Introduction

*COASY* protein associated neurodegeneration (CoPAN) is an autosomal recessive disorder caused by biallelic pathogenic mutations in the *COASY* gene (*COASY*, OMIM 609855) [1]. *COASY* encodes the mitochondrial bifunctional enzyme coenzyme A (CoA) synthase, which catalyses the final two steps in the pathway of intracellular CoA synthesis from pantothenic acid [2, 3]. Two broad *COASY* phenotypes have been described on a spectrum to date. Pontocerebellar hypoplasia type 12 (PCH12, OMIM 618266) is the severe foetal onset phenotype associated with microcephaly, pontocerebellar hypoplasia and parenchymal atrophy without evidence of excessive iron deposition on brain imaging [4]. The second phenotype, CoPAN, also known as neurodegeneration with brain iron accumulation type 6 (NBIA6, OMIM 615643), is characterised by variable childhood onset of progressive spasticity, dystonia, neuropathy, cognitive decline and neuropsychiatric features [1, 5] resulting from basal ganglia iron accumulation without a clear underlying pathogenic mechanism [1]. Brain magnetic resonance imaging (MRI) demonstrates features including thin corpus callosum, symmetric diffusion weighted imaging (DWI) abnormalities and T2 hyperintensity of the corpus striatum with patchy bilateral thalamic abnormalities [1, 5].

A previous report describes two siblings with CoPAN identified with newborn screening dried blood spot (DBS) acylcarnitine profiles resembling carnitine palmitoyltransferase 1a (CPT1) deficiency (CPT1, OMIM 255120) [5]. CPT1A is a mitochondrial outer membrane protein that reversibly converts long chain fatty acyl‐CoA species to their corresponding acylcarnitine moieties [6]. CPT1 deficiency is suggested on extended newborn screening via a characteristic pattern of elevated free carnitine (C0) and an elevated molar ratio of free carnitine to the sum of palmitoylcarnitine and octanoylcarnitine [C0/(C16+C18)] [6, 7]. This pattern is often reproducible on both DBS and plasma acylcarnitine profiles, although discrepancies between plasma and DBS free carnitine levels are recognised in CPT1 deficiency [8]. Whilst certain DBS acylcarnitine profile abnormalities require evaluation for several potential aetiologies, there are recent reports where DBS patterns of CPT1 deficiency had an alternative molecular diagnosis, with this being CoPAN [5].

We describe three patients who demonstrated a pattern of CPT1 deficiency on newborn screening DBS with subsequent normal plasma acylcarnitine profiles. All presented with or subsequently developed neurological abnormalities, and genetic studies identified pathogenic mutations in the *COASY* gene. This case series confirms that both *COASY* phenotypes can present with this DBS acylcarnitine pattern and CoPAN should be considered in the differential diagnosis of an abnormal extended newborn screening result suggestive of CPT1 deficiency.

## 2 Case Series

**Patient 1** was the first child to non‐consanguineous Caucasian parents. She was born at 37 weeks gestation via vaginal delivery in good condition, with APGAR scores of 9^1^ and 9^5^. Severe microcephaly was evident with a head circumference of 28 cm (< 1st centile). Birth weight was 2200 g (< 1st centile), and length 43 cm (< 1st centile). Physical examination demonstrated jitteriness, increased tone in limbs and brisk deep tendon reflexes. Nasogastric feeding was required from the first day of life due to a poor suck. Seizures presented on day one of life and remained refractory to anti‐epileptic drugs.

A DBS newborn screening card was collected on day four of life, and a subsequent repeat sample on day 6 of life, with the acylcarnitine profile demonstrating an elevated C0 and [C0/(C16+C18)] ratio, consistent with CPT1 deficiency (see Table 1). A plasma acylcarnitine profile collected on day 12 of life demonstrated a mild elevation in C0 but normal [C0/(C16+C18)], excluding CPT1 deficiency.

**TABLE 1:** Dried blood spot acylcarnitine profile results demonstrating pattern of CPT1 deficiency.

Patient	Age	C0 (μmol/L) (reference range 8–70 μmol/L)	C18:1 (μmol/L)	C18 (μmol/L)	C16 (μmol/L)	Ratio [C0/(C16+C18)] (reference range 2.5–45)
1	4 days	96	0.46	0.21	0.56	125
	6 days	78	0.53	0.16	0.62	96
2	3 days	100	0.44	0.16	0.5	151
	9 days	108	0.51	0.14	0.43	166
3	3 days	135	0.52	0.25	0.8	129
	49 days	76	0.56	0.21	0.42	130
	2 years	110	Not available	0.1	0.3	275

Abbreviations: C0, free carnitine; C16, palmitoylcarnitine; C18, octanoylcarnitine; C18:1, octadecenoylcarnitine.

**FIGURE 1:**
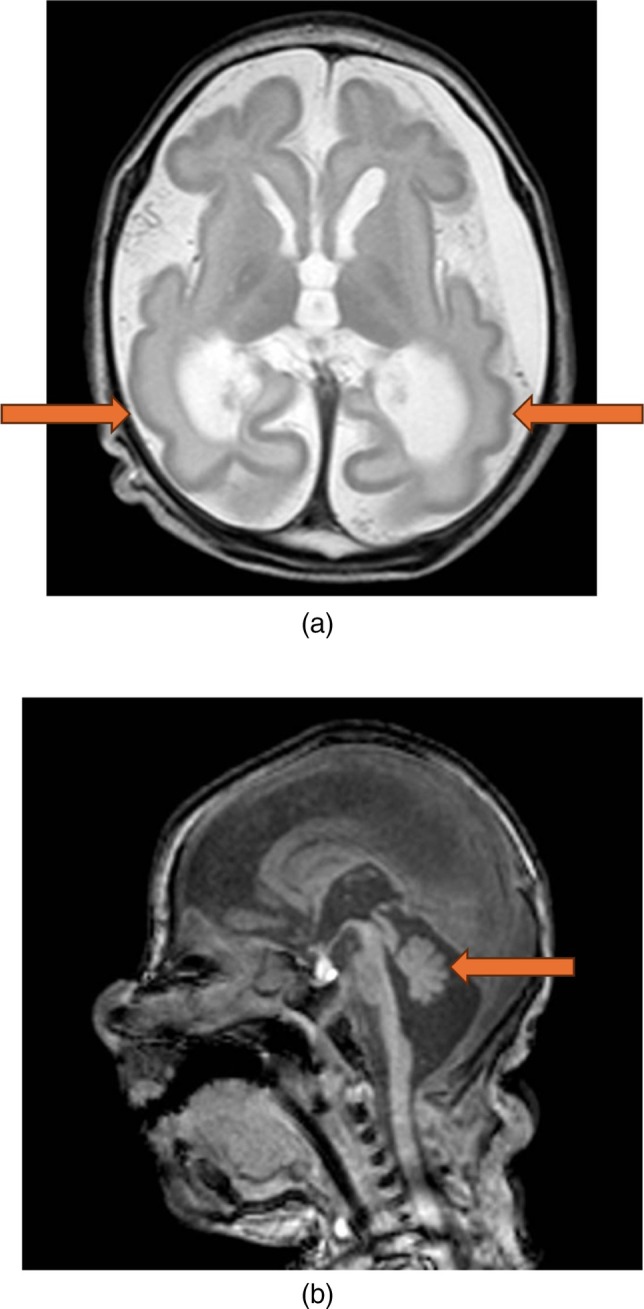
(a) T2 axial imaging showing diffuse parenchymal volume loss and simplified cortex. (b) T1 sagittal imaging showing pontocerebellar hypoplasia.

An electroencephalogram (EEG) on day 12 of life identified frequent subtle clinical and subclinical seizures. An MRI brain on day 15 of life demonstrated diffuse structural brain abnormalities including microcephaly, simplified gyral pattern, parenchymal atrophy, widened extra axial CSF spaces, dilated ventricles and pontocerebellar hypoplasia (see Figure 1a,b). Diffusion restriction was seen in the basal ganglia, thalami and there were bilateral subdural collections.

On day 17 of life, she developed necrotising enterocolitis. After initial successful management with antibiotics and cessation of enteral feeds, necrotising enterocolitis recurred on day 41 of life, with patient death on day 42 of life.

**Patient 2** was a dichorionic, diamniotic twin and a sibling of patient 1. Delivery was performed by caesarean section at 36 weeks due to poor foetal growth, with APGAR scores of 9^1^ and 9^5^. Microcephaly was evident, with a head circumference of 31.5 cm (< 1st centile). Birth weight was 2530 g (3rd centile) and length 46 cm (1st centile). Physical examination demonstrated increased limb tone and absent deep tendon reflexes. Like patient 1, nasogastric feeding was required from the first day of life due to poor suck. Drug resistant epilepsy also presented on the first day of life.

**FIGURE 2:**
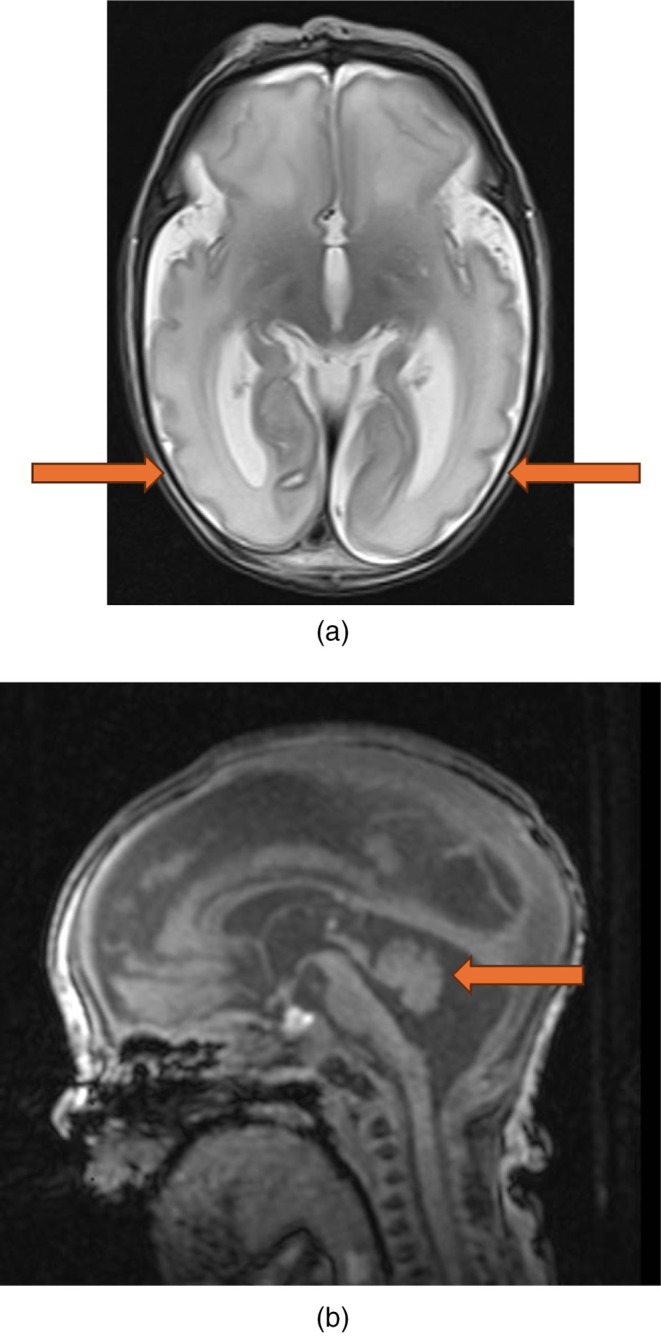
(a) T2 axial imaging showing simplified cortex. (b) T1 sagittal imaging showing pontocerebellar hypoplasia.

On day 2 of life, MRI brain identified diffuse structural brain abnormalities including microcephaly, simplified gyral pattern, parenchymal atrophy, widened extra axial CSF spaces, dilated ventricles and pontocerebellar hypoplasia (see Figure 2a,b). Finding were similar to patient 1 but of a much lesser severity.

A DBS card was collected on day 3 of life, with the DBS acylcarnitine profile demonstrating an elevated C0 and [C0/(C16+C18)], consistent with CPT1 deficiency (see Table 1). A repeat DBS card collected on day 9 of life demonstrated the same pattern of CPT1 deficiency with elevated C0 and [C0/(C16+C18)] (see Table 1). A plasma acylcarnitine profile performed on day 2 of life was normal, excluding CPT1 deficiency.

During infancy, the clinical course was characterised by a severe developmental and epileptic encephalopathy and drug resistant epilepsy. At age 17 months, feeding intolerance and recurrent intestinal pseudo‐obstructions developed, requiring intravenous parenteral nutrition. Further clinical deterioration ensued and the patient died at age 18 months. Posthumous whole exome sequencing performed at Victorian Clinical Genetics Service (VCGS) in the sibling pair identified pathogenic *COASY* mutations (c.1403_1404dupTG, paternal inheritance, ACMG class 5), (c.215A>G, maternal inheritance, ACMG class 4).

**Patient 3** was the first child of a nonconsanguineous Caucasian couple. Vaginal delivery occurred at 38 + 3 weeks following induction of labour for maternal gestational diabetes mellitus, with APGAR scores of 9^1^ and 9^5^. Birth weight was 2680 g (12th centile), head circumference was 33 cm (20th centile) and length was 48 cm (25th centile). On day 3 of life, admission to the special care nursery was required for phototherapy for unconjugated hyperbilirubinemia.

A DBS card was collected on day 3 of life, with the DBS acylcarnitine profile demonstrating an elevated C0 and [C0/(C16+C18)], consistent with CPT1 deficiency (see Table 1). A plasma acylcarnitine profile performed on day 12 of life demonstrated a mild elevation in C0 but normal [C0/(C16+C18)], excluding CPT1 deficiency. A repeat card was collected on day 49 of life, again demonstrating the CPT1 pattern of increased C0 and [C0/(C16+C18)].

At 5 months of age, she was diagnosed with gastroesophageal reflux and constipation. Mild motor developmental delay was apparent during infancy, with rolling at age 6 months and sitting without support achieved at 12 months.

**FIGURE 3:**
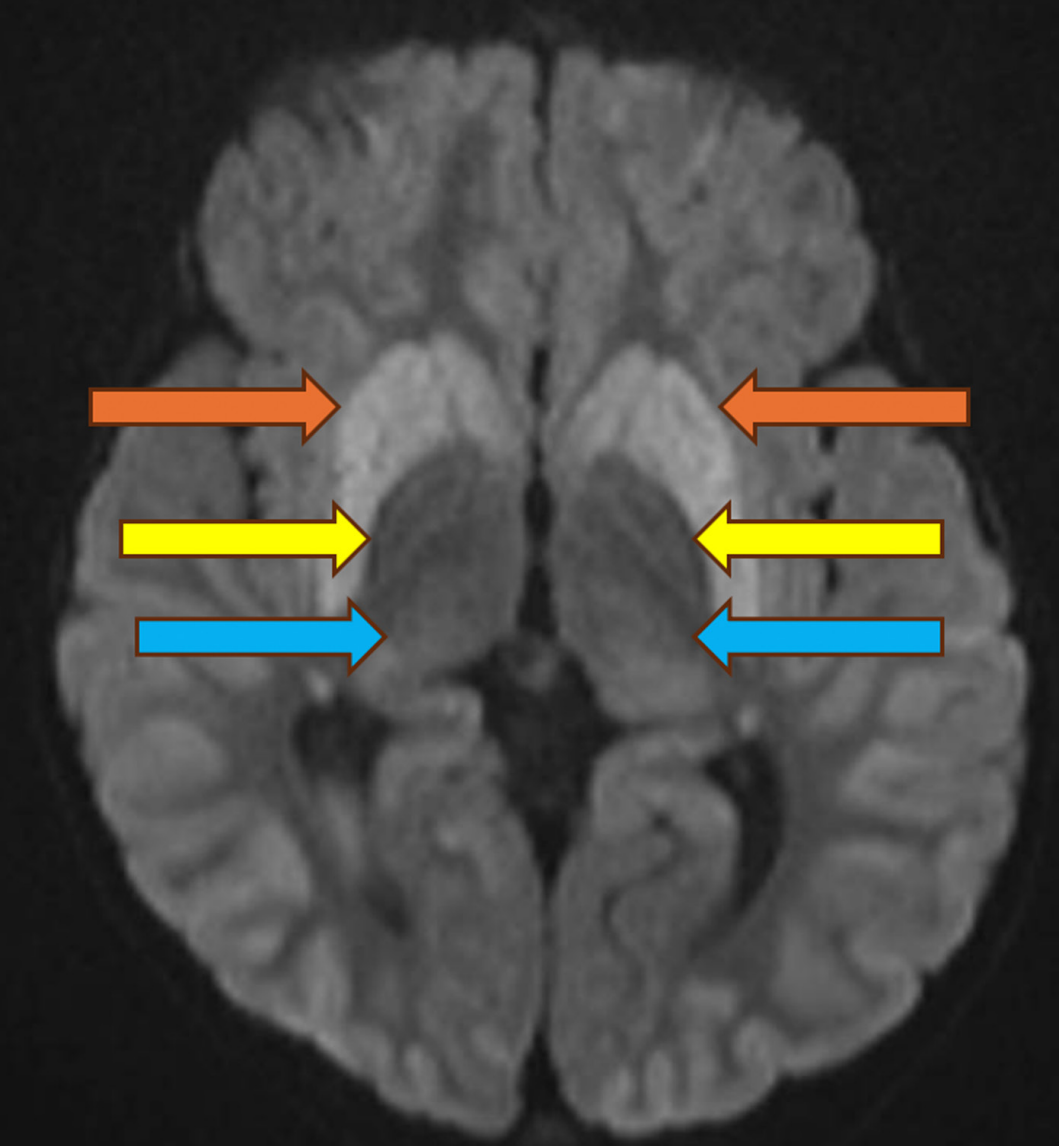
DWI demonstrating bilateral symmetric diffusion restriction in caudate nucleus and putamen (orange arrow), globus pallidus (yellow arrow) and thalami (blue arrow).

Her first focal seizure occurred at age 15 months. A computerised tomography (CT) brain was performed, demonstrating symmetric calcification in the globus pallidus. An MRI brain was performed at age 18 months demonstrating susceptibility artefact on susceptibility weighted imaging, a thin corpus callosum, symmetric T2 weighted imaging hyperintensity in the basal ganglia and thalami, and symmetric diffusion restriction in the cortex, basal ganglia and thalami (see Figure 3). Dystonia was present in the lower limbs, with evidence of brisk deep tendon reflexes in both upper and lower limbs.

Based on the DBS acylcarnitine profiles, clinical presentation and MRI features, a diagnosis of CoPAN was suspected. Whole exome sequencing performed at Fulgent Diagnostics demonstrated biallelic pathogenic mutations in *COASY* (c.1403_1404dupTG, ACMG class 4), (c.1495C>T, ACMG class 5). Parental segregation studies have not been performed to date.

## 3 Methods

### 3.1 Dried Blood Spot Acyclcarnitine Profile

In Queensland, DBS acylcarnitine profiles are performed by flow injection analysis tandem mass spectrometry. From 2004 to 2015, this was performed on a Waters quattro micro, and since 2015 on a Waters xevo‐TQD.

### 3.2 Next Generation Sequencing

Whole genome sequencing was performed on patient 2 at VCGS using massively parallel sequencing (Nextera TM DNA Flex Library Prep kit, illumine sequencers). Nuclear DNA had a mean target coverage of 30× and a minimum of 90% bases sequenced to at least 10×. Mitochondrial DNA analysis had a mean coverage of 800×. Variant analysis and interpretation were conducted within the selected target region using Agilent Alissa Interpret. There was insufficient specimen available for confirmatory genetic testing in patient 1.

Patient 3 underwent genomic testing at Fulgent Genetics using next generation sequencing technology. Phenotype‐related genes had 99.37% of target regions sequenced with at least 10× coverage of coding regions. Variants were interpreted manually using locus specific databases, literature searches and other molecular biological principles.

## 4 Discussion

This case series demonstrates a diagnosis of CoPAN present on newborn screening with a DBS acylcarnitine profile consistent with CPT1 deficiency. In our case series, all DBS samples collected in the neonatal and infantile periods had the pattern of CPT1 deficiency, with all plasma samples collected demonstrating a normal pattern. This pattern of abnormal DBS profiles and normal plasma acylcarnitine profiles is consistent with the pattern reported by Evers et al. in 2 siblings with CoPAN [5]. However, Stander et al. report a plasma acylcarnitine profile also consistent with CPT1 deficiency in 2 other siblings. The clinical and neuroimaging features of these reported cases are similar and summarised in Table 2.

**TABLE 2:** Clinical presentation and neuroimaging findings of reported cases.

Patient	Clinical presentation	Genotype (COASY gene)	MRI brain findings
1	Seizures	c.1403_1404dupTG c.215A>G	Microcephaly, simplified gyral pattern, parenchymal atrophy, widened extra axial CSF spaces, dilated ventricles and pontocerebellar hypoplasia. Diffusion restriction in the basal ganglia, thalami. Bilateral subdural collections.
2	Seizures	c.1403_1404dupTG c.215A>G	Microcephaly, simplified gyral pattern, parenchymal atrophy, widened extra axial CSF spaces, dilated ventricles and pontocerebellar hypoplasia. Findings were similar to patient 1 but of a much lesser severity.
3	Global developmental delay	c.1403_1404dupTG c.1495C>T	Thin corpus callosum, susceptibility artefact in the globus pallidi, symmetric T2 weighted imaging hyperintensity in the basal ganglia and thalami. Symmetric diffusion restriction in the cortex, basal ganglia and thalami.
4 (Evers et al.)	Hypotonia, global developmental delay	c.1495C>T; p.R499C c.C641T; p.A214V	Bilateral T2 hyperintensity and swelling of caudate nucleus, putamen and thalamus with associated restricted diffusion on DWI.
5 (Evers et al.)	Broad based gait, global developmental delay	c.1495C>T; p.R499C c.C641T; p.A214V	Bilateral T2 and FLAIR hyperintensity of caudate nucleus, putamen, thalamus and cortex.
6 (Rosati et al.)	Generalised hypotonia, minimal spontaneous movements	c.1664G>A; p.R555H	T2 hyperintensity of bilateral globus pallidi, thalami and posterior limbs of the internal capsule. Progressive atrophy bilateral cerebral hemispheres, basal ganglia and brainstem.
7 (Rosati et al.)	Generalised hypotonia, minimal spontaneous movements	c.1664G>A; p.R555H	T2 hyperintensity of posterior limbs of the internal capsule. Persistent symmetric abnormalities in diffusion restriction.
8 (Stander et al.)	Hypotonia, hyperglycaemia	Not available	Bilateral diffusion restriction in hippocampi, globus pallida, thalami, posterior limbs of internal capsule. Progressive atrophy consistent with pontocerebellar hypoplasia.
9 (Stander et al.)	Hypotonia, hyperglycaemia	Not available	Bilateral diffusion restriction in hippocampi, globus pallida, thalami, posterior limbs of internal capsule.

DBS acylcarnitine patterns resembling CPT1 deficiency are rare. Since 2004 in Queensland, more than 1 million infants have had DBS acylcarnitine screening, with only 4 patients identified with this profile. One patient had the mild Kiribati variant of CPT1 deficiency, with a plasma acylcarnitine profile consistent with CPT1 and identification of the pathogenic homozygous c.100C>Y mutation in *CPT1A*. The three remaining patients identified all had CoPAN.

CoA is an essential co‐factor in all living organisms and is required for several biochemical pathways including fatty acid metabolism and the tricarboxylic acid cycle [9]. Intracellular CoA is formed by a highly conserved five‐step pathway from pantothenic acid [10]. There are currently two known genetic disorders affecting enzymes in the CoA synthesis pathway: pantothenate kinase associated degeneration (PKAN, OMIM 606157), caused by pathogenic mutations in *PANK2*, and CoPAN [11]. In our case series, the c.1403_1404dupTG variant was present in all cases (although noting two patients were siblings). This is a nonsense variant predicted to lead to loss‐of‐function through truncated protein production [12].

The biochemical mechanism explaining the CPT1‐associated acylcarnitine abnormalities is not fully understood in CoPAN. Underlying mitochondrial dysfunction in these conditions may be an overlapping mechanism. A possible explanation is that defective *de novo* production of mitochondrial CoA results in the inability for fatty acids to be transferred from CoA to carnitine at the outer mitochondrial membrane. This theoretically could result in increased levels of free carnitine and decreased levels of long chain acylcarnitine species, resulting in the pattern of CPT1 deficiency. Evers et al. suggest this may lead to the biochemical abnormalities found on DBS [1]. However, there is currently no mechanism to explain the discrepancies between DBS and plasma acylcarnitine patterns in our cases of CoPAN. Acylcarnitine patterns are known to differ across tissue and body fluid types, which may be a contributor to this finding [13].

In pantothenate kinase associated degeneration (PKAN, OMIM 606157), fibroblast metabolomic studies demonstrated reduced levels of long chain acylcarnitine species. However, there have been no reports to date of patients with PKAN presenting on newborn screening with a pattern of CPT1 deficiency [14]. In a CoPAN mouse model, no significant reduction in CoA levels was measured on mass spectrometry of murine cerebrum compared with controls [15]. Fibroblast studies of two siblings with CoPAN also did not demonstrate a significant difference in measured levels of CoA compared with unaffected siblings.

## 5 Conclusion

In summary, this case series demonstrates that the phenotypic spectrum associated with pathogenic *COASY* mutations can present with the DBS acylcarnitine profile of CPT1 deficiency. A DBS pattern of CPT1 deficiency with a normal plasma acylcarnitine profile is suggestive of CoPAN and can be detected prior to the onset of neurological manifestations, identifying new potential for early diagnosis.

## Author Contributions

M.L. and S.M. prepared the original manuscript. All authors provided input into the manuscript. D.C. provided oversight. All authors take responsibility for the content of the article.

## Funding

The authors have nothing to report.

## Ethics Statement

Local human research ethics committee protocols were followed, including obtaining informed consent with a caregiver.

## Consent

Informed consent for preparation of this case report was obtained with a caregiver for each case subject.

## Conflicts of Interest

The authors declare no conflicts of interest.

## Data Availability

The data that support the findings of this study are available on request from the corresponding author. The data are not publicly available due to privacy or ethical restrictions.
